# Surface density of polyarginine influence the size, zeta potential, cellular uptake and tissue distribution of the nanostructured lipid carrier

**DOI:** 10.1080/10717544.2016.1269849

**Published:** 2017-02-09

**Authors:** Mingshuang Sun, Zhihong Zhu, Huixin Wang, Cuiyan Han, Dandan Liu, Lei Tian, Xinggang Yang, Weisan Pan

**Affiliations:** 1 School of Pharmacy, Shenyang Pharmaceutical University, Shenyang, China,; 2 School of Pharmacy, Qiqihar Medical University, Qiqihar, China, and; 3 School of Biomedical & Chemical Engineering, Liaoning Institute of Science and Technology, Benxi, China

**Keywords:** Poly-arginine, nanostructured lipid carriers, surface density, cellular uptake, tissue distribution

## Abstract

Poly-arginines are strong tools to elevate the cellular uptake of nanopreparations. To learn the influence of poly-arginine (RRRRRRRR, R8) density on a series of properties of nanostructured lipid carrier (NLC), we build six R8 modified NLCs with different R8 densities (nR-NLC, where n represents the R8 ratio) by fusion–emulsion method with the aid of stearyl-R8. The pharmaceutical characteristics like size, zeta potential and *in vitro* drug release, cellular uptake, cytotoxicity to A549 cells and tissue distribution in S180 tumor-bearing mice of the six nR-NLCs are all investigated. It turns out that with as little as 2% weight ratio of stearyl-R8 modified on NLC, its pharmaceutical properties, especially zeta potential changes astonishingly; however, the stearyl-R8 ratio should be higher than 4% to upgrade the cellular uptake and cytotoxicity evidently; in the *ex vivo* tissue distribution assessment, the nR-NLC with less than 8% R8 showed similar tissue accumulation, while NLC with 10% R8 shows obvious acute toxicity to mice. Our study pays attention to the effect of the R8 ratio on the changes of cargo properties, and the results indicate that this topic is essential and worth to be further developed.

## Introduction

It is not surprising that the discovery of short peptides, which can translocate through cell membranes and transfer large biologically active molecules inside the cells, has attracted much attention in the field of drug delivery. These peptides are generally known as the cell-penetrating peptides (CPPs). Ever since discovered in the 1980s, more CPPs are found, synthesized, and evaluated including Tat, Pnetratin, VP22, and poly-arginine (Trehin & Merkle, 2004; Pujals & Giralt, 2008; Gautam et al., 2015; Ye et al., 2015).

Poly-arginines are among the most frequently utilized CPPs, not only for the intracellular delivery of proteins, but also for the delivery of various molecules including magnetic beads, RNAs, and even nanocarriers (Futaki et al., 2001; Khalil et al., 2006; Khalil et al., 2008; Kim et al., 2010; Park, 2012; Zhang et al., 2015). They are artificial peptides synthesized according to the arginine-rich structure of the Tat protein (Futaki et al., 2001; Futaki, 2005). Poly-arginines bear net positive charges because of the guanidine group in the monomer, and the electrical property is often considered in the judgment of whether they have been modified on the cargo surface because the zeta potential of the nanocargo would turn to positive when poly-arginines are successfully modified (Nakamura et al., 2007; Kang et al., 2014). Although they are artificial peptides, poly-arginines exhibit similar or sometimes stronger cell penetrating ability than Tat peptide which is a typical CPP, and Futaki has proved that the cellular uptake of nonamer of arginine is 20 times faster than Tat at 37 °C (Futaki, 2005).

Poly-arginines with around 8 monomers are the most popular ones in the poly-arginine family. Researchers have reported that in the *in vitro* cellular uptake study, the cellular uptake speed and amount elevate with the increase of polymerization degree (with polymerization degree between 4 and 16, R4 and R16); however, after the intravenous administration to mice, R8 turns out to be the one which accumulates most in the tumor tissue. Moreover, its accumulation in tumor tissue is even higher than that in heart, liver, and other healthy tissues (Park, 2012). Similar outcomes occur in the experiment of cellular uptake of polyarginine conjugated with carbonic anhydrase with different R8 chain lengths (Futaki et al., 2001). Such results have put R8 on the forefront of the poly-arginine family, and its capability of mediating cargos into the cells is repeatedly proven (Oyarzun-Ampuero et al., 2011; Lozano et al., 2013; Alhakamy et al., 2016).

Nanostructured lipid carriers (NLCs) are promising new-generation colloidal lipid carriers in the antitumor drug delivery system. The limitations of solid lipid nanoparticles (SLN), including limited drug loading, gelation, and drug leakage during storage due to lipid polymorphism, have been shown to be overcome by NLC (Tiwari & Pathak, 2011; Garg et al., 2016). As NLC contain both solid lipid and liquid lipid, various ingredients could be used in the NLC formula including glyceryl monostearate, stearic acid, Compritrol 888 ATO R, etc. (solid lipid), and Soybean Oil, oleic acid, medium chain oil, etc. (liquid lipid), and many other lipid ingredients not to say various emulsifying agents, including Poloxamer 188, Lecithin and other productions like the Kolliphor series of BASF Co. Ltd (Zhang et al., 2008, 2014; Han et al., 2014; Garg et al., 2016). The modification of functional moiety on NLC is sometimes accomplished by the conjugation of functional moiety with lipid materials to form amphipathic materials since most of the functional moieties are hydrosoluble (Zhang et al., 2006; Torchilin, 2008; Kim et al., 2010).

Many groups have proved that the modification of R8 could elevate the intracellular transportation of nanopreparations, however, limited researches focus on the influence of R8 density on the nanopreparation properties (Khalil et al., 2008), which we consider is essential because it is widely recognized that R8 has astonishing capability of assisting nanopreparations to enter the cells, but the issues about whether there is an optimum concentration, a lowest effective concentration or even a highest effective concentration are still open to discuss. Moreover, it has been reported that the R8 peptide could accumulate in the tumor tissue despite that CPPs lack tumor specificity (Park, 2012), we are curious about whether R8-decorated NLC could cumulate in tumor tissue and the impact of R8 density on the tissue distribution. In the present manuscript, we prepared six R8-modified NLCs with different R8 density (the R8 concentration was 0%, 2%, 4%, 6%, 8% and 10% weight ratio to the total lipid weight, and the preparations were recorded as nR-NLC in which the ‘n’ represents the concentration percentage), and the pharmaceutical characteristics were evaluated; in addition, the cellular uptake was calculated and observed, besides, the anticancer drug paclitaxel was loaded into nR-NLC and the cytotoxicity to A549 cells was studied. In addition to the systematic *in vitro* investigation, we have explored the tissue distribution of Dir-loaded nR-NLC in S180 tumor-bearing mice. We consider this study significant because it provides important information on how to choose the CPP density for particular use.

## Materials and methods

### Materials

Stearyl-polyarginine (STR-R8) with greater than 98% purity was synthesized by TeraBio Technology Co., Ltd. (Guangzhou, China). Glycerin monostearate (GMS), Kolliphar ELP, and Kolliphor HS15 were generously presented by BASF Co., Ltd. (Ludwigshafen, Germany). Medium chain triglyceride (MCT) was purchased from Yuhao Chemical Co., Ltd. (Hangzhou, China). Comarin 6 (Cou 6) was purchased from J&K Scientific Ltd. (Shanghai, China). Paclitaxel (PTX) was purchased from Jiangsu Hengrui Pharmaceutical Co., Ltd. (Jiangsu, China). DiR iodide (1,1′-dioctadecyl-3,3,3′,3′-tetramethylindotricarbocyanine iodide) was purchased from Fanbo Co., Ltd. (Beijing, China).

Non-small cell lung cancer cell A549 cell was purchased from the Type Culture Collection of the Chinese Academy of Sciences (Shanghai, China). The cells were cultured in RPMI-1640 culture medium (Hyclone Co., Ltd., Thermo Fisher Scientific., Cramlington, UK), supplemented with 10% fetal bovine serum (Sijiqing Co., Ltd., Hangzhou, China). When the cells grew to 80–90% confluence, they were trypsinized and passaged in the ratio of 1:3. All other chemicals and reagents were of analytical or cell culture grade.

### Methods

#### Preparation and pharmaceutical properties of nR-NLC

nR-NLCs were prepared by the fusion–emulsification method (Zhang et al., 2013). Briefly, Compatrol ATO 888, GMS, MCT, Kolliphor ELP and different amount of STR-R8 (2%, 4%, 6%, 8% and 10% weight ratio to the total lipid) were mixed and melted at 80 °C to get the uniform and transparent oil phase; Kolliphor HS15 was dispersed in double-distilled water at the same temperature to get the aqueous phase which was then added into the oil phase dropwise with constant stirring. After that, the emulsion was cooled immediately at 4 °C to get the nR-NLC. 0R-NLC was set to be the controlled group in all the following studies, and its preparation was as above only with no STR-R8 in the formula.

Model drug PTX or fluorescent material Cou6 or Dir was mixed with the oil phase when preparing PTX-loaded nR-NLC or Cou6-loaded nR-NLC or Dir-loaded nR-NLC. The mean diameter, particle size distribution and zeta potential of nR-NLC were all learned by Malvern Zetasizer Nano ZS90 (Malvern, UK), each sample was diluted by distilled water for 50 times before test.

To learn the *in vitro* drug release of PTX-loaded nR-NLC, dialysis method was employed. Briefly, 50 ml PBS (pH 7.4) containing 0.5% Tween 80 was chosen to be the drug release medium (Tsai et al., 2013). nR-NLC was placed in the dialysis bag (MW 8∼14KDa) with being sealed. The dialysis bag was immersed in the release medium and shaken at 100 rpm and 37 °C. At predicted time points (2, 4, 6, 8, 24, 36, and 48 h), 1 ml of release medium was taken out and an aliquot of fresh medium was supplemented. The samples were assayed by HPLC and the accumulative drug release was calculated.

#### Quantitative analysis of the cellular uptake of nR-NLC

A549 cells were seeded in the 24-well plate at the density of 1 × 10^5^ cells/well and incubated overnight to allow the cell attachment, the culture medium was then replaced by FBS-free fresh RPMI-1640 culture medium containing Cou6-loaded nR-NLC at the Cou6 concentration of 2 μg/ml. Further incubation for 0.5, 1, 2 and 4 h was conducted at 37 °C, after which the culture medium was discarded and the cells were rinsed with PBS for 3 times. 100 μL RIPA Lysis Buffer (Beyotime, Shanghai, China) was added into each well to lyse the cells, and 25 μL lysates were taken out to determine the protein content in each well using BCA Protein Assay Kit (Beyotime, China). The fluorescence intensity of the residue was measured by a Microplate Reader (Thermo Scientific, Waltham, MA), and the intracellular Cou6 amount was calculated and divided by the protein content to obviate the error caused by the cell amount difference. The cellular uptake of nR-NLC at various incubation times was obtained and compared.

The cellular uptake of nR-NLC at 4 h at 4 °C were also investigated, the process was the same with the quantitative assay only with the temperature changed to 4 °C.

#### Confocal laser scanning microscopy (CLSM) observation

The cellular uptake process of nR-NLC was the same as in Section “Quantitative analysis of the cellular uptake of nR-NL”; after the incubation time period, the cells were washed with PBS for 3 times and fixed with 4% paraformaldehyde for 20 min, and stained by DAPI for the nuclei. The cells were observed with CLSM (TCS SP5 II, Leica; LSM710, CarlZeiss, Oberkochen, Germany).

#### Cytotoxicity of nR-NLC

A549 cells were seeded in the 96-well plate at the density of 1 × 10^4^ cells/well and incubated overnight to allow the cell attachment. At the second day, the cells were treated with the growth medium containing blank nR-NLC or PTX-loaded nR-NLC for another 24 h, then 10 μl MTT solution (5 mg/ml) was added into each well and incubated for another 4 h. Then, the medium was replaced by 200 μL DMSO to dissolve the formazan crystals and the absorbance was measured at 490 nm. Cells treated without any preparations were set to be the controlled group. The relative cell viability was calculated by Equation (1):

(1) $$\mathrm{Relative}\;\mathrm{cell}\;\mathrm{viability}\;\left(\%\right)=A_{\mathrm{tested}}/A_{\mathrm{controlled}}\times 100\%$$

#### Tissue distribution of Dir-loaded nR-NLC in S180 tumor-bearing mice

Dir was encapsulated in nR-NLC to assay their tissue distribution in S180 tumor-bearing mice. Previous study has guaranteed the good stability of nR-NLC in the blood plasma at 24 h. After the preparation of Dir-loaded nR-NLC, free Dir was removed using the filter membrane with the aperture of 200 nm due to its minimum solubility in water.

The S180 cells were passaged by the ascitic fluid in Kunming Mice, the third passage of the ascetic fluid was diluted once and injected to the left flank of the mice, the tissue distribution experiment was conducted on the 7th day after tumor cell injection.

S180 tumor-bearing mice were divided into six groups with six mice included in each group, after the injection through the tail vein with Dir-loaded nR-NLC (the Dir concentration was 2.5 mg/kg), three mice in each group were sacrificed at the predicted time point (6 and 24 h), tumor and other tissues were collected and the pictures of which were taken using the *In Vivo* FX PRO imaging system (Bruker, Karlsruhe, Germany). The demands of the Animal Ethics committee have been carefully followed in the experiments related with mice.

## Results

### Pharmaceutical properties of nR-NLC

The mean particle diameter, polydistersity index (PDI), and zeta potential of nR-NLCs are listed in Table 1. The average size of the preparations from 0R-NLC to 10R-NLC increased from 50.81 to 88.00 nm with acceptant PDI smaller than 0.5. Zeta potential of nR-NLC also exhibited an uplifting trend, the minimum R8 density at 2% could cause as many as +18 mv augment, and the value of the parameter continued to elevate as the R8 amount increased. But, one should notice that when the R8 density reached 6%, further increase in R8 density led to the zeta potential increment drop.

**Table 1.** Pharmaceutical characteristics of nR-NLC.

Parameters	0R-NLC	2R-NLC	4R-NLC	6R-NLC	8R-NLC	10R-NLC
Size (nm)	50.81 ± 3.42	52.09 ± 2.88	63.87 ± 2.65	68.2 ± 3.13	70.31 ± 2.98	88.00 ± 4.02
PDI	0.223 ± 0.012	0.309 ± 0.044	0.306 ± 0.023	0.322 ± 0.025	0.296 ± 0.031	0.487 ± 0.022
Zeta potential (mv)	−4.21 ± 0.26	13.90 ± 0.12	18.61 ± 0.08	24.84 ± 0.15	25.72 ± 0.15	26.33 ± 0.44

Each parameter was measured in triplets. The results are presented as mean ± SD.

**Figure 1.**
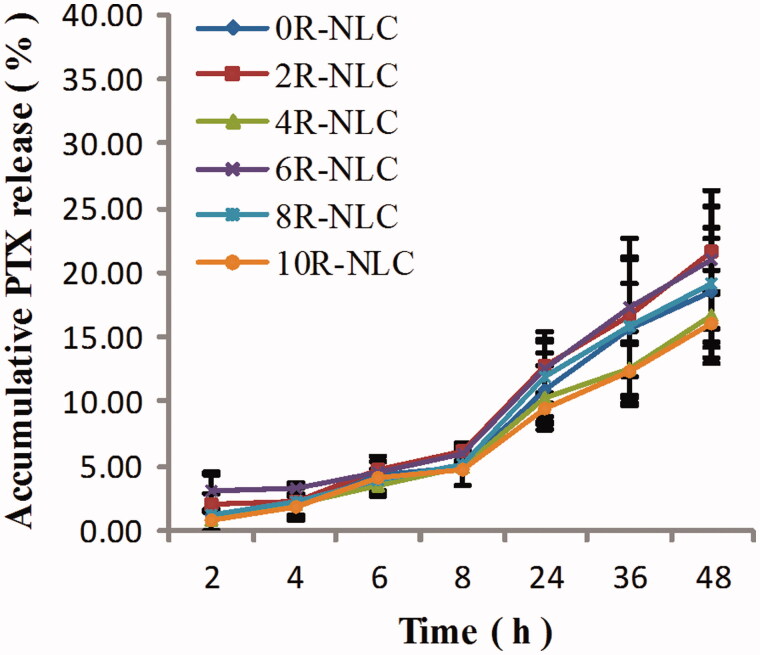
*In vitro* drug release of PTX-loaded nR-NLC.

All the six nR-NLCs showed sustained drug release profile as listed in Figure 1, the accumulative drug release in 48 h were all below 30%. As the influence of R8 modification on the drug release was hard to read from the figure, the *in vitro* dissolution similarity factor (f2) was calculated using 0R-NLC as the reference product with Equation (2) (Xie et al., 2015), and the f2 of 2R-NLC to 10R-NLC compared with 0R-NLC were 87, 88, 86, 98 and 85, respectively; as the f2 were all above 50, we could say that the various decoration amount of R8 showed no influence on the drug release activity.

(2) $$f2=50\times \mathrm{lg}\;\left[\frac{100}{\sqrt{1+\frac{\sum_{i=1}^{n}{\left(\mathrm{Rt}-\mathrm{Tt}\right)}^{2}}{n}}}\right]$$

where *n* is the total amount of the time point, *Rt* was the drug release of 0R-NLC at certain time point, *Tt* was the drug release of other nR-NLC at certain time point.

**Figure 2.**
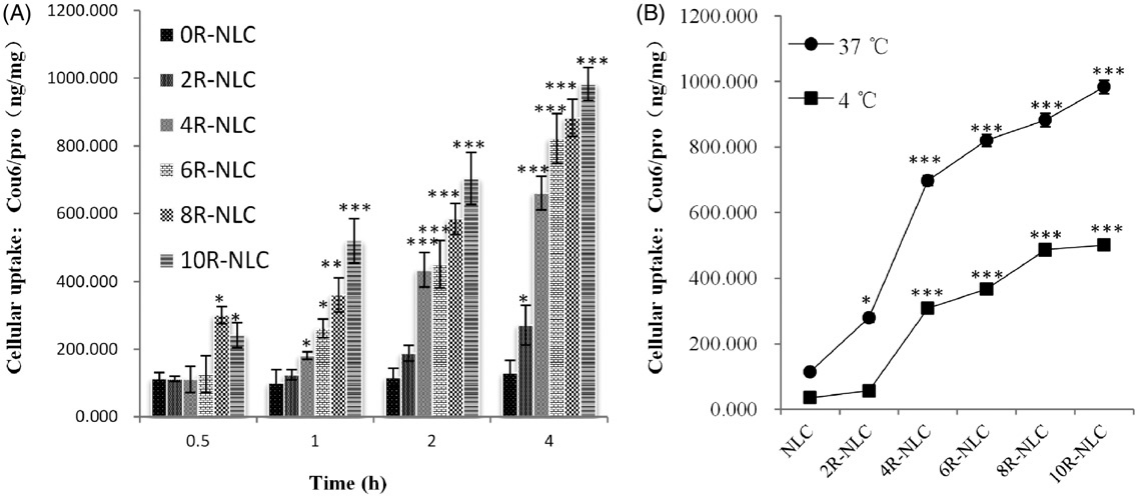
Cellular uptake of nR-NLC by clearage method. (A) The cellular uptake of Cou 6-loaded nR-NLC by A549 cells at different incubation time at 37 °C, the Cou 6 concentration was at 2 μg/ml. (B) The cellular uptake of Cou6-loaded nR-NLC at 37 °Cand 4 °C at 4 h, the Cou6 concentration was at 2 μg/ml. Results are presented as mean ± SD (*n* = 3). Significant differences from 0R-NLC are indicated as follows: *p < 0.05, **p < 0.01 and ***p < 0.001.

### Cellular uptake and cytotoxicity

The results of *in vitro* cellular uptake by A549 cells are shown in Figure 2(A). For a specific nR-NLC, the cellular internalization increased as time prolonged; however, the augment varied as the R8 amount differed, the augment was relatively low for 0R-NLC and 2R-NLC, the internalization at 4 h were 1.15 and 2.41 times of 0.5 h, respectively; however, the elevation of 4R-NLC to 10R-NLC was much astonishing as they showed nearly 5 times augment at 4 h to 0.5 h (8R-NLC showed 2.93 times). The influence of R8 density on the cellular uptake could be observed more clearly when analyzing the cellular uptake of nR-NLC under the same incubation time. At 0.5 h, the cellular uptake of 2R-NLC to 6R-NLC showed no significant improvement compared with 0R-NLC (*p* > 0.05), while 8R-NLC and 10R-NLC exhibited obvious elevation (*p* < 0.05). At 1 h, compared with 0R-NLC, the effect of 2R-NLC was still not significant (*p* > 0.05), but 4R-NLC to 10R-NLC performed the elevation effect in various degrees. At 2 and 4 h, the cellular uptake of 2R-NLC was higher than 0R-NLC (*p* < 0.05), but the effect was still weaker than the other groups.

**Figure 3.**
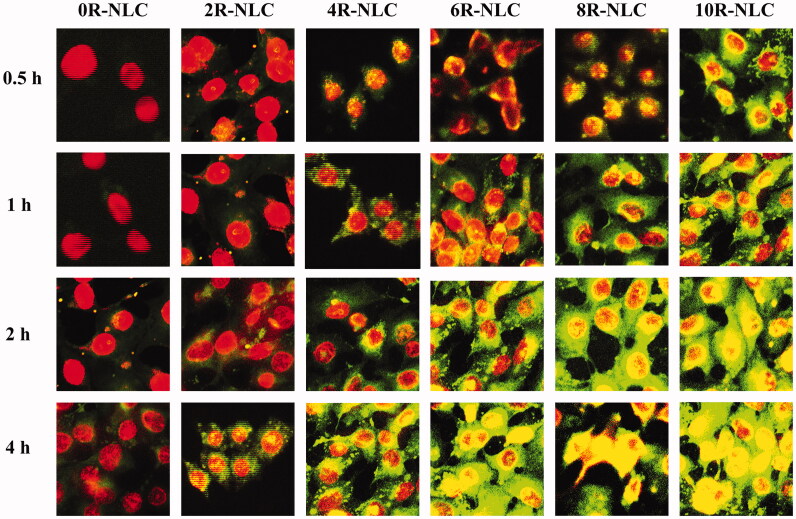
Cellular uptake of Cou 6-loaded nR-NLC in A549 cells at different incubation time. The Cou 6 concentration was 2 μg/ml. The pictures were taken by CLSM (TCS SP5 II, Leica; LSM710, CarlZeiss). The cell nuclei was stained by DAPI.

It could be directly read from Figures 2(A) and 3 that at 2 and 4 h, 4R-NLC served as the boundary, and the groups at the right hand performed extremely higher cellular uptake than 0R-NLC. Taking 4 h as an example, the cell internalization of 4R-NLC to 10R-NLC were 5.07, 6.31, 6.78 and 7.55 times of 0R-NLC while the value was only 2.08 times for 2R-NLC. The results mentioned above have vividly indicated that the R8 density has key impact on the internalization of the cargo, thus for the purpose of faster and more cellular uptake, the R8 density should be at least higher than 4%. We have also tested the cellular uptake of Cou 6 loaded nR-NLC by the method of flow cytometry, and results were listed in supplementary materials which showed similar tendency with the present manuscript.

Figure 2(B) indicates the cellular uptake of nR-NLC at 37 °C and 4 °C, it could be concluded that the low temperature suppressed the internalization of nR-NLC, this result suggested that the nR-NLC internalization involved with energy; interestingly, similar to that at 37 °C, 4R-NLC still served as the boundary as the cellular uptake of the groups at the right hand of it were still a lot higher than 0R-NLC.

**Figure 4.**
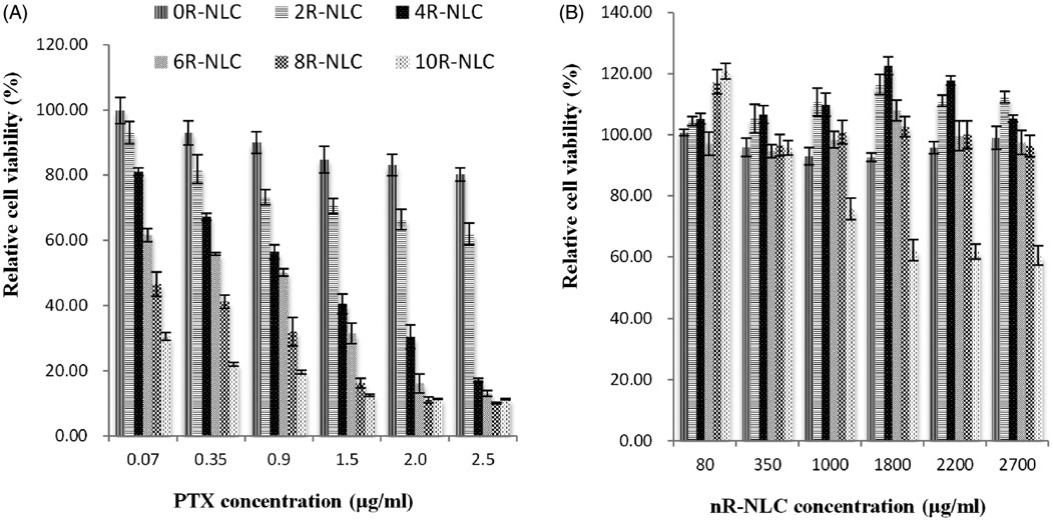
*In vitro* cytotoxicity of nR-NLC in A549 cells at 24 h. (A) The cytotixicity of PTX-loaded nR-NLC at various PTX concentration; (B) The cytotoxicity of blank nR – NLC, the concentration was in accordance with (a). Results are presented as mean ± SD (*n* = 6). The legend is identical in the pictures.

The cytotoxicity of PTX-loaded nR-NLC to A549 cells is listed in Figure 4(A), at the same PTX concentration, the cytotoxicity increased as the R8 density elevated, which trend was in accordance with the cellular uptake. But, we were more curious about whether the cell suppression effect was caused by the nR-NLC cargo since the drug release of PTX from the nR-NLC was very slow. Figure 4(B) has answered the question, except for the blank 10R-NLC which showed cytotoxicity at concentrations higher than 1000 μg/ml, other nR-NLC exhibited no suppressed effect to the cells. These findings suggested that the cytotoxicity to the cells were all caused by the PTX that entered the cells except that in the 10R-NLC group, where the suppression was caused by the combined effect of the 10R-NLC cargo and PTX.

### Tissue distribution of nR-NLC

**Figure 5.**
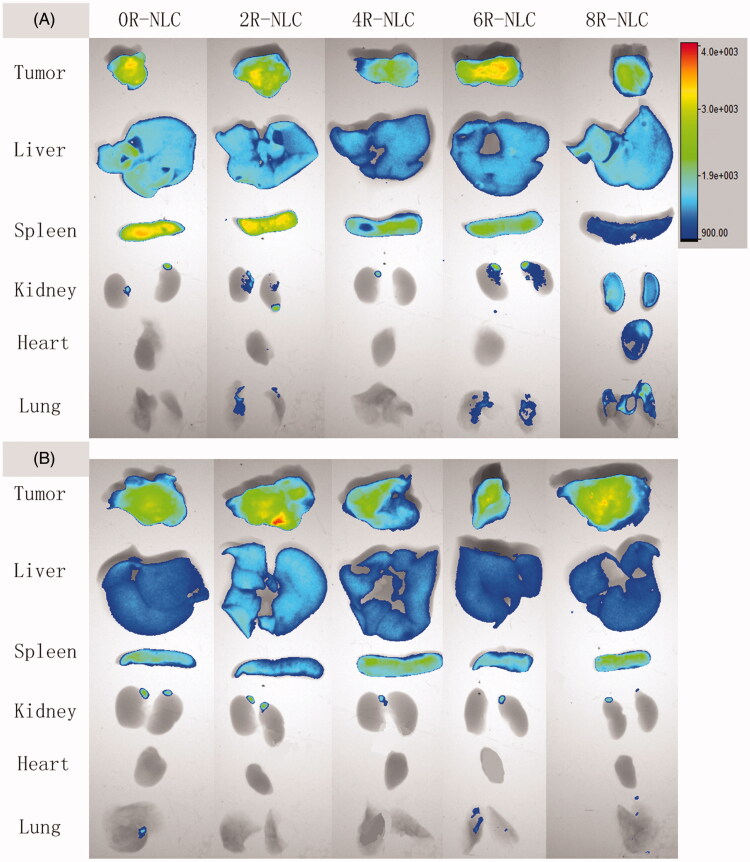
Tissue distribution of Dir-loaded nR-NLC in S180 tumor-bearing mice. The Dir concentration was 2.5 mg/kg. The organs were collected after the sacrifice of the mice, and the pictures were taken by the *In Vivo* FX PRO imaging system (Bruker, Germany) with the same exposure time, the minimum and maximum fluorescent intensity was 900 and 4000, respectively, in all the groups.

The *ex vivo* tissue distribution of nR-NLC in S180 tumor-bearing mice is shown in Figure 5. As shown in the figure, 6 h after the intravenous injection, nR-NLC (except for 8R-NLC) showed similar tissue distribution, besides the apparent accumulation in tumor tissue which we believe was contributed by the EPR effect, un-negligible fluorescent signal appeared in the blood abundant tissues like liver and spleen; nevertheless, 8R-NLC performed more like a systemic distribution, the fluorescent signal in spleen was weaker than the other groups while obvious signals was seen in heart, lung, and kidney. However, when the time prolonged to 24 h, the distinctness dismissed as all the tested nR-NLC accumulated in tumor, liver and spleen, and their strength were nearly identical. What was worth noticing was that about 10 min after the administration of 10R-NLC, the mice showed an arched back and standing hair indicating they were suffering pain, so we sacrificed them according to the Demands of the Ethics Committee.

## Discussion

It has been a widely accepted scientific fact that upon modification with CPPs the pharmaceutical properties, cellular uptake and even *in vivo* tissue distribution of nanoparticles would alter. However, studies on the influence of CPPs density on these properties were few. So in this study, we investigated on this topic using R8 as the model CPP hoping to provide information on choosing the CPP density for various purposes.

In the pharmaceutical properties, theoretically, the increase of R8 on the NLC surface would not change the size significantly since R8 was only several nanometers long, besides, it should be the elevation of the covering degree instead of the size as the R8 amount increased; however, our results suggested that the average size of nR-NLC increased as R8 amount amplified. We believe that when preparing nR-NLC, the more STR-R8 added to the oil phase meant the more lipid in the preparation since the stearyl group also attributed to lipid material, so the ratio of emulsifier to total lipid became lower, which lead to an elevation of the particle size (Joshi et al., 2012). In addition, since the nR-NLC particle would attract cationic ions due to the guanidino groups, there would be a “corona”-like structure around the nR-NLC particle. When measured by DLS, it was the construct composed of nR-NLC and the “corona” that diffract the incident light rather than the pristine nR-NLC (Bhattacharjee, 2016). In this case, more R8 decoration meant thicker “corona”, which also affected the results of the size measurement. In contrast to the variation of average size, the elevation of zeta potential was more in line with our expectation. When applied to an electric field, nR-NLC would attract ions to form the electric double layer (EDL), the electric potential at the slipping plane in the EDL was the so-called zeta potential (Bhattacharjee, 2016). In the present study, many cationic particles were absorbed to the EDL of nR-NLC due to the strong attraction of guanidine groups in the R8 structure, so theoretically, zeta potential would be elevated as R8 density increased. However, in reality, zeta potential would not be amplified without eternity due to the limited ion amount in the solution and the restricted distance that the electrostatic effect of nR-NLC could extend, which we believe was the reason why when the R8 density reached 6%, further increase in R8 density led to the zeta potential increment drop. Drug release profile as another important pharmaceutical property was of great significance in the evaluation of an antitumor drug delivery system; generally, a sustained drug release profile was necessary because it could maintain less drug exposure to healthy tissues and more drug enter the tumor tissue along with the nanopreparation via EPR effect. The results in our group indicated that the variation of R8 amount would not change the sustained drug release profile of nR-NLC. Although the f2 has suggested that the R8 density has little influence on the drug release of nR-NLC, the accumulative drug release at 48 h exhibited difference. Obviously, there was no clear rule in the effect of R8 density on this drug release parameter. In fact, the dissolution profile of NLC related to many factors like the compositions, structures, the preparing temperature, the solidifying temperature, size, the surfactant being used and even the viscosity of the matrix (zur Mühlen et al., 1998; Müller et al., 2002). As for PTX-loaded nR-NLC, the impact of the added STR-R8 on the interaction of PTX with oil phase and aqueous phase was unpredicted; besides, after the addition of STR-R8, the ratio of emulsifier to total lipid changed resulting in the alter of the nR-NLC structure; moreover, the size of the nR-NLC also affected the drug release behavior. All the factors related to each other and mutually restrict each other resulting in the irregular change of the accumulative drug release at 48 h.

Another important aim of the present study was to investigate the influence of R8 density on the cellular uptake. It could be concluded that different R8 density should be selected for various purposes, but generally, to obtain an evident cellular internalization elevation effect, the R8 density should not be lower than 4%. Moreover, the cellular uptake at 4 °C gave the reason why over 4% R8 modified nR-NLC showed faster cellular uptake. Apparently, when R8 amount exceeded 4%, nR-NLC tended to enter the cells through some energy-independent pathways. It has been declared that this phenomenon related to the hydrophobic counter-anion pyrenebutyrate on the cell membrane formed a hydrophobic complex with arginine-rich CPP and neutralized their positive charge to assist direct enter to the cells (Guterstam et al., 2009).

Although outstanding capacity on mediating nanoparticles internalization to cells has been performed, one reason that hindered the extensive use of R8 was its absence of cell specificity which could arouse significant risk when used *in vivo*. R8-modified nanoparticles were believed to accumulate in blood abundant tissues like heart, liver, and spleen, which may cause potential unwanted toxicity (Park, 2012). Our results have vividly demonstrated that when the R8 density was less than 8%, the tissue distribution of the pristine carrier could not be changed. Nevertheless, when considered to be used *in vivo*, the R8 density should not be higher than 8% because 8% R8 modification alters the tissue distribution. In addition to the tumor, liver, and spleen accumulation, the un-negligible accumulation in lung, heart, and kidney indicated potential toxic risk, when the R8 density increased to 10%, the uncomfortable behavior of the animal suggested sever acute toxicity. However, other groups have declared that no obvious toxicity was observed when the injected dose of R8 reached as high as 5 mg/kg (Suhorutsenko et al., 2011), thus we speculate that when R8-modified nanoparticles were used *in vivo*, the properties of the nanoparticle should also be carefully evaluated. Besides, we have to admit that our results also indicated that despite the varied R8 density, the tumor accumulation remained almost the same, such finding contradicted with the *in vitro* cellular uptake study. According to literature, we believe that despite the pristine surface of nR-NLC, when circulated in the body, nR-NLC interacted with biomacromolecules in the blood to form the so-called “protein corona”, which made the “nR-NLC” that the body cell saw was totally different from the pristine ones (Lynch et al., 2009), we assume that in the present study, the protein corona has surface properties similar to nR-NLC to the same level which led to similar tumor accumulation *in vivo*.

## Conclusion

We have constructed six STR-R8-modified nR-NLCs, and the decorated concentration was 0%, 2%, 4%, 6%, 8% and 10% weight ratio to the total lipid. We have found that the R8 density has obvious impact on their size and zeta potential, these two parameters were elevated as R8 density increased; besides, it was proved that the R8 density variation would not alter the drug release profile of nR-NLC. In the *in vitro* cellular assays, to obtain distinctive effect, the R8 concentration should be at least 4% weight ratio to the total lipid, while to obtain faster effect, higher than 8% was necessary. Besides, what contradicted with popular recognition was that our results proved that with less than 6% R8 modification, the tissue distribution behavior would not alter, but when the R8 concentration reached higher than 8%, the tissue distribution started to change and 10% modification would cause severe acute toxicity which would even cause animal death.

## Supplementary Material

supplemental_datas.docx

S_Figure_1..tif

## Notes

**Supplementary material available online**
