# Supplementary material for: Surface density of polyarginine influence the size, zeta potential, cellular uptake and tissue distribution of the nanostructured lipid carrier
Source: Drug Deliv. 2017 Feb 9;24(1):519–26. doi: 10.1080/10717544.2016.1269849 (PMC8253139; doi:10.1080/10717544.2016.1269849)
Supplement: supplemental_datas.docx [file IDRD_A_1269849_SM5609.docx]

We have also tested the cellular uptake of nR-NLC at different time points by the method of flow cytometry (presented in Supplemental Figure 1). The results have indicated similar tendency with the method used in the manuscript that different surface density of R8 strongly affect the cellular internalization, and at least 4% R8 density was necessary for effective performance of R8.

Besides, we have also measured whether the R8 internalization into the cells would alter the total cell protein concentration since this parameter was essential for the results. After 4 h incubation with 0 - 100 μg STR-R8, A549 cells gave out nearly identical total protein concentration at around 0.6 mg/ml (P>0.05), such outcoming have supported the cleavage method being used in the manuscript.

Although flow cytometry and cleavage method both gave out similar cellular uptake tendency, the reason we choose the cleavage method was it represented the cellular uptake amount in the mass irrespective of the single cells and the results were more easily to be compared to each other.

Supplemental Figure 1. Cellular uptake of nR-NLC by A549 cells measured by the flow cytometry at different time points. (a) 0.5 h; (b) 1 h; (c) 2 h; (d) 4 h.
